# Development of an Evaluation System for Transfer Care Skills Using Embroidered Body Pressure and Proximity Sensor

**DOI:** 10.1109/JTEHM.2023.3294062

**Published:** 2023-07-10

**Authors:** Hirofumi Kurosaki, Hiromu Shirahata, Junya Kawahara, Yasuhito Kondo, Ken Kondo, Bumsuk Lee, Masato Odagaki

**Affiliations:** Graduate School of Maebashi Institute of Technology Maebashi 371-0816 Japan; Gunma Industrial Technology Center Maebashi 379-2147 Japan; Faculty of RehabilitationGunma Paz University Takasaki 370-0006 Japan; Faculty of Medicine School of Health SciencesGunma University 12925 Maebashi 371-0044 Japan

**Keywords:** Caregiver skills, motion analysis, nursing care, textile sensor, transfer motion

## Abstract

Objective: It is important to improve caregiving skills to help reduce the strain on inexperienced caregivers. Previous studies on quantifying caregiving skills have predominantly relied on expensive equipment, such as motion-capture systems with multiple infrared cameras or acceleration sensors. To overcome the cost and space limitations of existing systems, we developed a simple evaluation system for transfer care skills that uses capacitive sensors composed of conductive embroidery fibers. The proposed system can be developed with a few thousand US dollars. Method: The developed evaluation system was used to compare the seating position and velocity of a care recipient during transfers from a nursing-care bed to a wheelchair between groups of inexperienced and expert caregivers. To validate the proposed system, we compare the motion data measured by our system and the data obtained from a conventional three-dimensional motion-capture system and force plate. Results: We analyze the relationship between changes in the center of pressure (CoP) recorded by the force plate and the center of gravity (CoG) obtained by the developed system. Evidently, the changes in CoP have a relation with the CoG. We show that the actual seating speed ( $v_{\mathrm {z}}) $ measured by the motion-capture system is related to the speed coefficient calculated from our sensor output. A significant difference exists in $v_{\mathrm {z}}$ between the inexperienced group and the physical therapists/occupational therapists’ group. Conclusions: The proposed system can effectively estimate a caregiver’s skill level in transferring patients from a bed to a wheelchair in terms of the seating position and velocity.

***Clinical and Translational Impact Statement—*** To alleviate the burden on caregivers in clinical environments, we developed a novel sensor utilizing conductive fibers and demonstrate its capability to assess caregiving skills. The proposed evaluation system can effectively evaluate the nursing care skills of various caregivers in medical and nursing care settings at a low cost and small space for equipment.

## I. Introduction

In the caregiving industry, physical stress is a prevalent problem for care providers, who are often required to assist with labor-intensive activities, such as lifting, transferring, and repositioning individuals with mobility limitations. Performing such strenuous activities regularly over a long period can lead to long-term physical strain and injury, particularly if caregivers are not adequately trained in safe lifting techniques or lack necessary equipment. In particular, transferring patients is physically demanding for a caregiver and can cause health issues, such as back pain and work-related musculoskeletal disorders (MSDs), which are a common concern for healthcare workers. There is evidence suggesting that frequent patient transfers increase the risk of MSDs [1], and lower physical exposure of healthcare workers during transfers has been linked to lower odds of low back pain [2]. Furthermore, inadequate caregiver skills can result in numerous incidents, such as falling out of bed.

A method for quantitatively evaluating caregiver skills is necessary to alleviate the burden on caregivers and reduce the risk of accidents by enhancing their caregiving abilities.

Several studies have evaluated physical conditions during transfers, including sitting movements by the Wheelchair Skills Training Program (WSTP); however, the evaluation relied on subjective third-party observation methods [3], [4].

Previous research has suggested the use of motion-capture cameras [5], [6] or wearable motion-capture systems [7] for evaluation. Several studies have investigated the impact of different transfer caregiving methods on the pressure distribution on wheelchair seat surfaces [8], [9]. Buisseret et al. [10] used acceleration sensors to evaluate caregiver skills; however, the sensors provided complicated and insufficient information about the evaluation of caregivers’ skills. Moreover, these methods have not been widely adopted because of the size and cost constraints of the devices.

Regarding the seating position on a wheelchair during transfer, Requejo et al. and Redford et al. reported that a slouched posture was associated with sacral sitting and sliding out of the wheelchair, which can cause shearing type pressure ulcers [11], [12]. Pedersen et al. reported that a wheelchair back, which supports the seated spinal curves, improves upright posture, functional reach, and wheelchair propulsion skills [13].

Thus, it is crucial to evaluate the seating position during transfer to prevent patient injury. Previous studies [8], [14] have revealed that the transfer motion induced by non-experts cause greater contact pressure for the simulated patient when seated, narrowing the surface area of the seat and forcing the patient’s seating position to become shallow. When training under clinical settings, the buttocks of a patient sitting in a wheelchair should be seated as deeply (inwardly) as possible. In a shallow seating position, the pelvis tends to tilt backward, resulting in a so-called “slouching posture”. If the patient needs assistance in transferring from a wheelchair to a bed, it is necessary to help the patient sit as deeply as possible. Enhancing the caregiver’s technique and knowledge can help position a patient in an optimal seating position, reducing contact pressure and potentially preventing adverse effects on the care recipient, such as pressure ulcers.

Iwakiri et al. reported the evaluation of lifting and lowering velocities while using a patient lift for transfer during nursing care [15]. They assessed how the care recipient felt during transfer motion. The appropriate transfer velocity using a lifting device was evaluated by measuring the heart rate (HR) and electromyography (EMG). It was concluded that the recommended velocity for lowering the resident is 50 mm/s within the range of 30–90 mm/s. Thus, there exists an appropriate seating velocity to ensure the comfort of the patient. Regarding the safety aspect in preventing accidents, for a relatively fast velocity, the caregiver may feel cautious and have an increased HR and muscle tension. To ensure safety, ISO 10535 (2006) [16] mandates that the velocity of lifting and lowering via a patient lift should not exceed 15 mm/s when loaded and 25 mm/s when unloaded.

**FIGURE 1.**
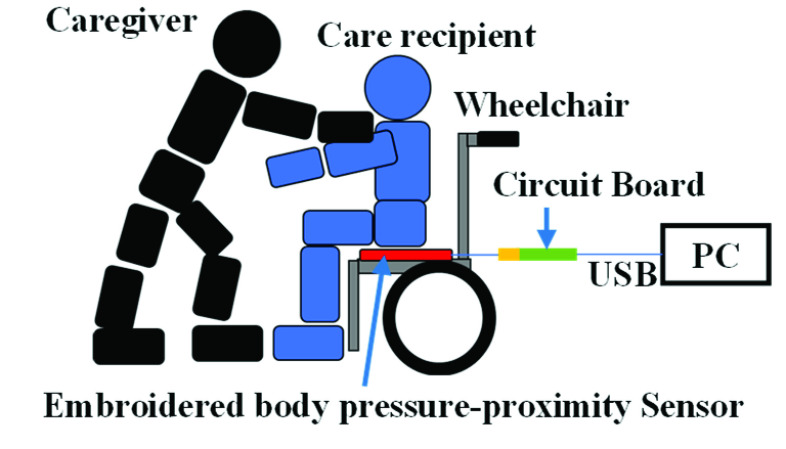
Overview of the transfer skill evaluation system.

Based on these reports, we propose a simple evaluation system that employs multi-channel body pressure and proximity sensors composed of conductive embroidery fabrics to measure the seating velocity and seating position of a care recipient during transfer. In our previous studies, we demonstrated the capability of conductive textile sensors to measure the seating position of a chair [17] and detect the seating velocity by assuming the device will be used in nursing care [18]. However, we did not investigate the difference of proficiency of the caregiver in transfer motion. Fig. 1 shows the system overview which comprises an embroidered body pressure-proximity sensor with a measurement circuit board and measurement PC. The capacitive sensors in the proposed system measure the pressure on the seated body of the care recipient and the distance between their buttocks and the seat of the wheelchair in seating or standing assistance.

Compared to a conventional motion sensing system, two features are distinct in the proposed system: (1) low cost and (2) small space, primarily because of the simple structure of conductive fibers embroidered on the felt fabric. Moreover, our system can be developed with a few thousand dollars, whereas commercial motion capture systems cost hundreds of thousands of dollars. Additionally, our system does not require a large space, such as an experimental room for equipment of the motion capture cameras; instead, the sensor array is placed on the wheelchair.

To assess the validity of the proposed system, we compare the seated position and velocity of care recipient during transfers from a nursing-care bed to a wheelchair between inexperienced and expert groups. To validate our system, we compare the motion data collected by our system with data obtained using a commonly employed three-dimensional motion-capture system and a force plate. We aim to determine whether our system can accurately evaluate the skill level of a caregiver in transferring patients from a bed to a wheelchair, specifically in terms of the seating position and velocity.

## II. Methods and Procedures

### A. Embroidered Capacitive Body Pressure and Proximity Sensor

The development of pressure sensing structures is a growing area of research involving textile-based sensors. There are several techniques to produce textile-based pressure sensors using capacitive sensors, resistive sensors, piezoelectric materials, and conductive particles within compressible structures.

X. Ye, M. Tian et al. developed a fabric-based wearable capacitive sensor with pressure detection. The sensor can detect human motion once it is attached to the body surface but it is not applicable for body proximity [19]. Wijesiriwardana et al. fabricated capacitive fiber-meshed transducers for touch and proximity-sensing applications [20]. The developed sensor was intended for applications of touch sensing as a wearable interface, thus both proximity and pressure characteristics were not shown. To measure the body pressure, e-textile pressure sensors based on conductive fiber and its structure has been proposed [21], and the sensor system could measure the body pressure at 3,960 points; however, it was not adopted to evaluate human motion. For the application of motion analysis by a textile pressure sensor [22], it could successfully provide measurements for gait analysis by a socks-type sensor. It would be useful for the walking function, but it is not suitable for evaluating the caregiver skill as the number of sensors is low and the center of pressure is not shown while walking. We demonstrated a textile sensor that can measure both the body pressure and amount of sweat for detecting pressure ulcers [23]. However, none of them demonstrated the capability of proximity and pressure sensing using e-textiles. Therefore, we developed a novel capacitive fabrics sensor using embroidered technology and succeeded in precisely measuring the proximity and pressure characteristics [17], [18].

The novelty of the system lies in its application as a motion sensor for evaluating a caregiver’s skill, despite the simple sensor structure. None of the existing sensors can effectively detect the position and velocity of a patient in transfer motion. This concept brings significant innovation to the field of caregiving and aged care, particularly in terms of usability. The sensor array used in the evaluation system is not sewn into the wheelchair seat and can be easily removed. Our design allows for the sensor to be washable or disposable, ensuring hygiene and convenience in clinical settings.

**FIGURE 2.**
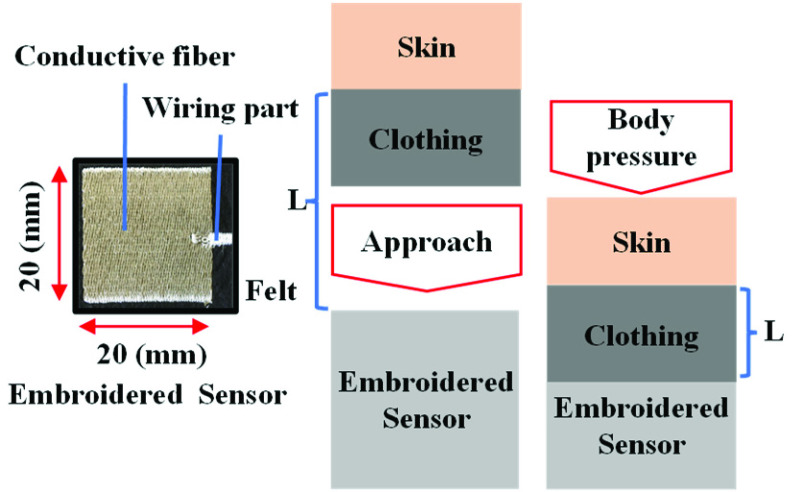
Embroidered capacitive body pressure and proximity sensor.

We designed a simple sensor composed of conductive embroidery fibers to measure the gap between the wheelchair seat and the buttocks of a person, as depicted on the left side of Fig. 2. The sensor component consists of a single-pole electrode composed of conductive fibers (Smart-X, Fujix Ltd., Japan), which are embroidered onto felt in 20 mm squares. The wiring part is coated with conductive fibers and insulated with an embroidery thread. The electrode generates a parasitic capacitance when it is in open space. When a person gets close to the electrode, a parallel plate capacitance is formed, which is mathematically described in (1).

\begin{equation*}C = \varepsilon _{0}\kappa \frac {S}{L}. \tag{1}\end{equation*}

The formula used to calculate the capacitance, as shown in (1), includes factors such as the distance $L$ between the body and the conductive fiber (mm), dielectric constant of vacuum $\varepsilon _{0}$ at $8.854\times 10^{lpzrptMinus12}$ (F/mm), relative permittivity of clothing $\kappa $, and the area of the embroidered conductive fiber $S$ in square millimeters. As a person moves toward the sensor, the distance between the person and conductive fiber decreases, which increases the capacitance. This change in distance over time can be measured and is referred to as the approach velocity. Upon reaching and contacting the sensor, the body pressure of the person compresses their clothing, further reducing the gap between the body and the sensor, resulting in an increase in capacitance, which enables body pressure measurement. As mentioned above, it is assumed that the sensor can measure the velocity of the seating motion and body pressure after being seated.

### B. Evaluation System for Caregiver Skills

**FIGURE 3.**
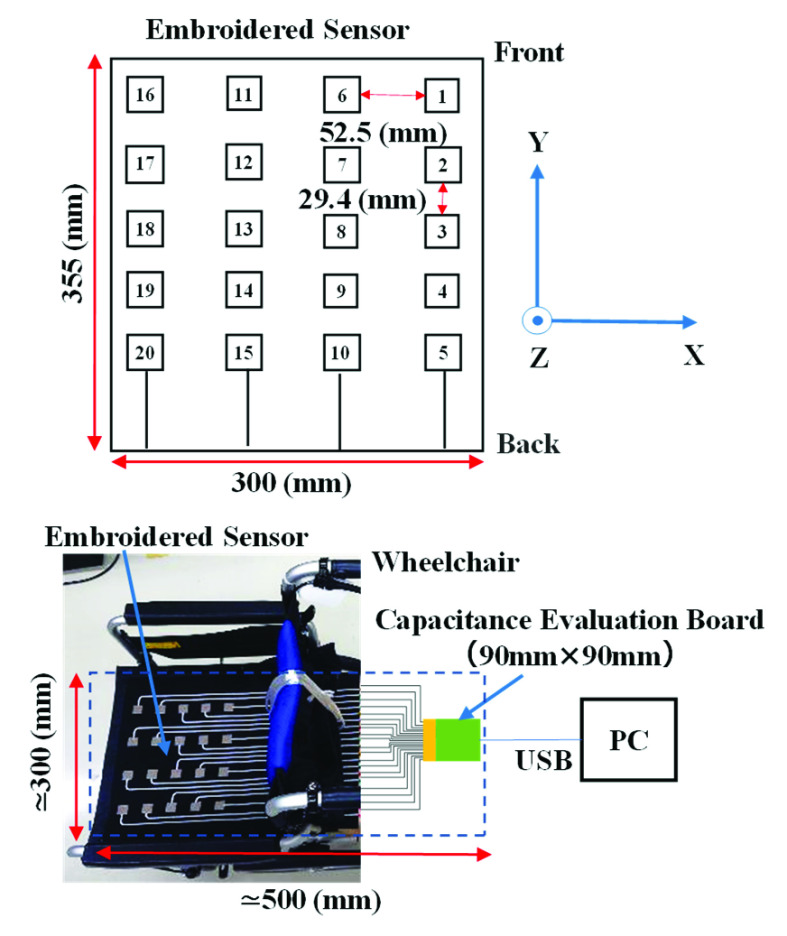
Sensor layout and evaluation system with developed sensors.

Fig. 3 shows the placement of the 20-channel embroidered capacitive body pressure and proximity sensors on the wheelchair seat in a $4\times5$ arrangement. The size of the sensors is approximately 355 mm $\times300$ mm. The sensor outputs are linked to a capacitance evaluation circuit board (RX130 Touch Sensor Evaluation Kit, Renesas Electronics Corporation, Japan) equipped with a built-in switched-capacitor filter. The size of the circuit board is 90 mm $\times90$ mm. The overall size of the system is approximately 500 mm $\times300$ mm, excluding the laptop PC. The changes in capacitance detected by the embroidered sensors were recorded by a PC via a serial communication interface at a sampling frequency of 62 Hz. The capacitance variations between the body and conductive fibers are transformed into changes in the electrical current via a switched-capacitor filter. The changes were evaluated by counting the signal pulses generated by a circuit that modulates the oscillation frequency based on the magnitude of the current. The resulting count value reflects the changes in capacitance caused by the close proximity or contact with the body. The right, forward, and upward directions correspond to the positive x-axis, positive y-axis, and positive z-axis, respectively, of the seat coordinate system.

Generally, several cameras are installed around the subject of motion analysis when performing motion analysis using a motion capture system. Thus, a space of approximately 5 m $\times5$ m is required for covering the range of human motion. In contrast, as shown in Fig. 3, the proposed system needs a relatively smaller space than a motion capture system.

### C. Determination of Seating Position With Center of Gravity (CoG)

**FIGURE 4.**
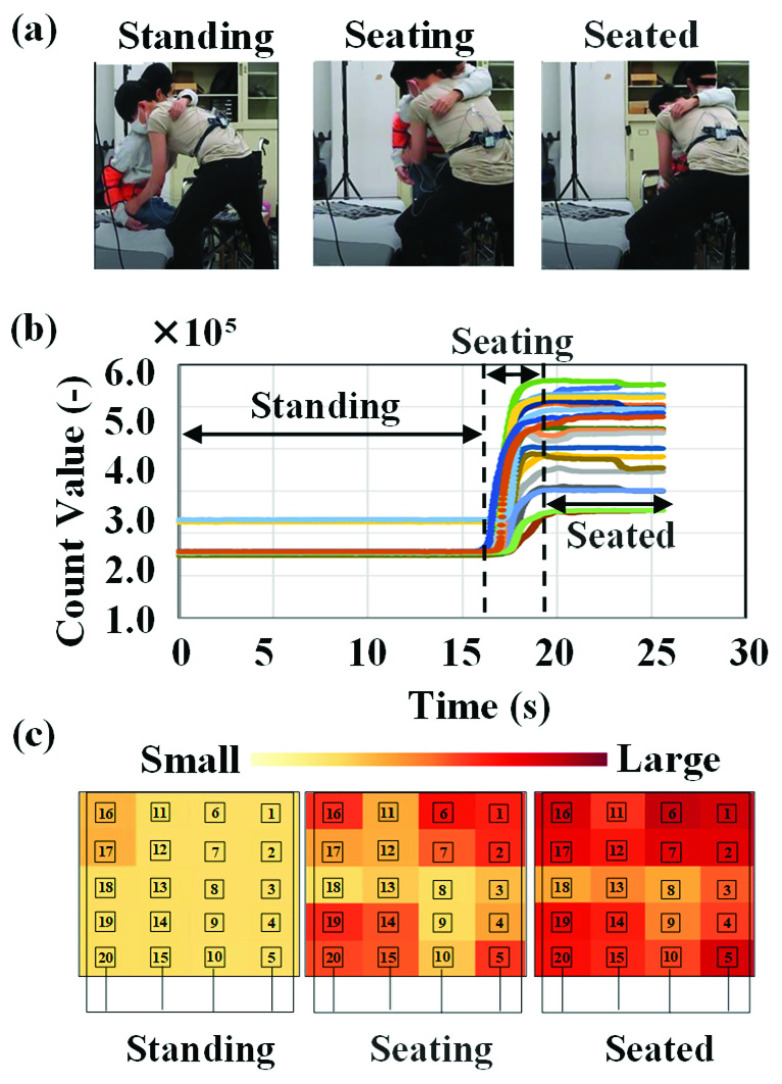
Sensor response during transfers from bed to wheelchair. (a) Three phases of the transfer from bed to wheelchair: standing, seating, and seated. (b) Transition waveforms of the count values; the horizontal and vertical axes represent time (s) and count values, respectively. (c) Heat map of the count values in each phase with low and high areas indicated in yellow and red, respectively.

Fig. 4 (a) depicts three distinct phases of transferring from a bed to a wheelchair: standing, seating, and seated. Fig. 4 (b) displays the transitional waveforms of the count values, where the horizontal and vertical axes represent time (s) and the count value, respectively. Fig. 4 (c) illustrates a heat map of the count values in each phase with low and high areas shown in yellow and red, respectively.

As depicted in the figure, the outputs from all sensors increase rapidly during the approach phase and then stabilize in the seating phase. We suggest that the seating position and velocity can be determined by analyzing the data from the body pressure and proximity sensors, which can then be used to evaluate the transfer proficiency of the caregiver. The center of gravity in the x- and y-directions (CoG_x_ and CoG_y_) of the sensor output was calculated using (2) and (3).

\begin{align*} {\mathrm {CoG}}_{\mathrm {x}}\left ({\mathrm {t} }\right) = \frac {\sum \nolimits _{i = 1}^{20} {cnt_{i} \left ({t }\right) x_{i}} }{\sum \nolimits _{i = 1}^{20} {cnt_{i} \left ({t }\right)}} \tag{2}\\ {\mathrm {CoG}}_{\mathrm {y}}\left ({t }\right)= \frac {\sum \nolimits _{i = 1}^{20} {cnt_{i}\left ({t }\right) y_{i}} }{\sum \nolimits _{i = 1}^{20} {cnt_{i} \left ({t }\right)}}. \tag{3}\end{align*}

The CoG values were calculated using a 10-second period of sensor data collected prior to the patient completing the seating process and evaluated based on the position (mm) relative from the origin, represented by $x_{i}$ and $y_{i}$, and the count value of each sensor at time $t$. The count value of each channel is represented as $cnt_{i} $(t). Thus, CoG_x_ and CoG_y_ are zeros if the patient is seated at the center of the seat.

**FIGURE 5.**
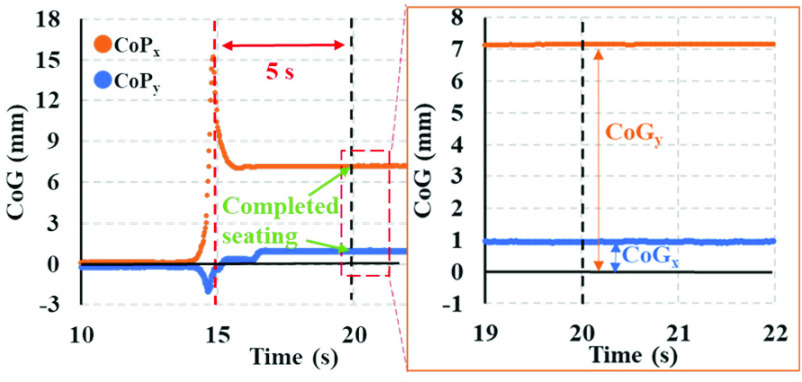
Calculation of CoG as an indicator of the seating position.

Fig. 5 illustrates the waveform of the center of gravity (CoG) during one experiment. The horizontal and vertical axes represent the time in seconds and CoG value, respectively. Before the patient approaches the wheelchair seat, both CoG values (CoG_x_ and CoG_y_) are almost zero. However, as the patient approaches the seat, the CoG values start to change. Seating was considered completed at five seconds after CoG_y_ reached its maximum value, and at this point, the CoG values become stable. The data enable us to verify the proper seating position of the patient.

### D. Determination of Seating Velocity

**FIGURE 6.**
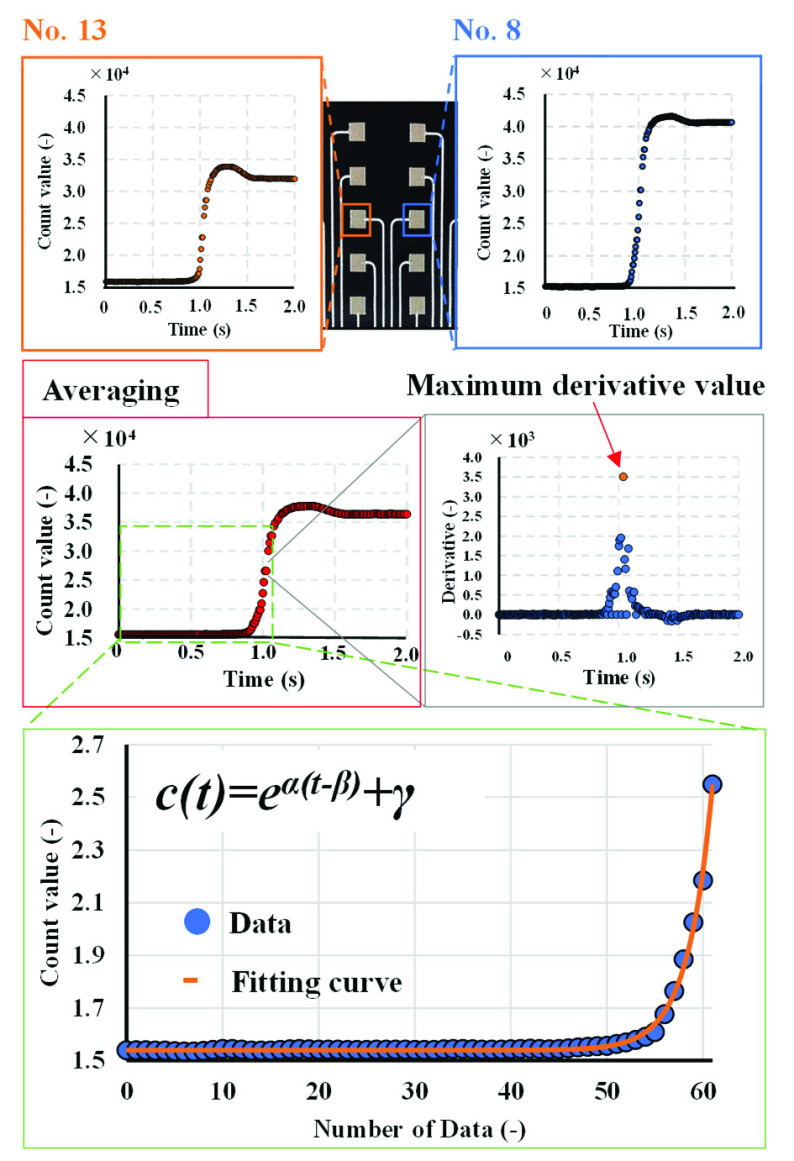
Calculation of the velocity coefficient $\alpha $ as an indicator of the seating velocity.

The computation of the seating velocity from the sensor output data involves determining the velocity at which the care recipient descends to the seat. Fig. 6 illustrates the mapping of the sensor responses to the fitting curve. The mapping was achieved by utilizing the average waveform obtained from two sensors designated as channel number 13 and 8 (as shown in Fig. 3). The waveform depicts the output data of the sensors over time, providing valuable insights into the acceleration and deceleration of the monitored patient. The fitting equation is defined in (4), with the count function containing an exponential term.

\begin{equation*} c \left ({t }\right) = e^{\alpha \left ({t-\beta }\right)} + \gamma, \tag{4}\end{equation*}

where c(t) represents the count value at a specific time $t$, $\alpha $ is a parameter that reflects the velocity of seating and indicates the rate of increase of the exponential term, $\beta $ denotes the time at which the sensor output begins to increase, and $\gamma $ symbolizes the asymptotic value of the exponential term. Data corresponding to one second (62 data points) prior to the appearance of the maximum derivative in the sensor response were used for fitting, as shown in the plot at the bottom of Fig. 6.

### E. Evaluation of System Validity

#### 1) Experimental Setup

**FIGURE 7.**
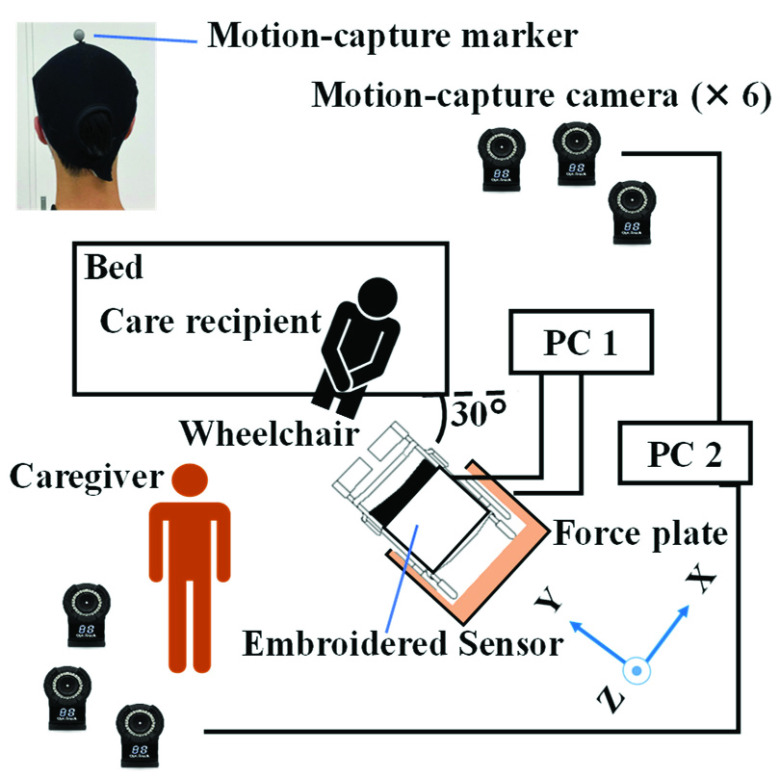
Experimental setup for system validity.

The experimental setup is illustrated in Fig. 7. The aim of the experiment was to assess the proficiency of a nursing caregiver during transfer from the bed to the wheelchair. The experimental setting was set up with a care bed and wheelchair angled at 30°. The body pressure and proximity sensors, which are capacitive-embroidered sensors, were placed on the wheelchair seat, and data were collected using a computer (PC 1). To validate the system, the motion data obtained from the system were compared with data obtained from a three-dimensional motion-capture system (OptiTrack, Acuity Inc., Japan) and a force plate (CFP60XS302UIO, Shintokogio Ltd., Japan). A motion-capture system was used to monitor the seating velocity, and data were recorded on a separate computer (PC 1). A reflective marker was placed on top of the care recipient’s head for tracking, and the force plate was positioned beneath the wheels of the wheelchair to confirm the seating position. The motion-capture system had a sampling rate of 120 Hz, and the velocity was measured and recorded using PC 2. The force plate had a sampling rate of 600 Hz, and the center of pressure (CoP) was measured and recorded using PC 1. The axial direction of the force plate was aligned with the coordinate system of the capacitive-embroidered body pressure and proximity sensors.

#### 2) Participants and Experimental Procedure

Three groups (care recipient, inexperienced participants, and physical therapists/occupational therapists (PT/OTs)) of participants were selected: one care recipient in the first group, eight inexperienced participants in the second group, and six PT/OTs in the third group. The demographic information for each group of participants is presented in Table 1. The care recipient was a 24-year-old healthy male (height: 163 cm, weight: 50 kg, right-handed, no motor disorders) who wore a right hemiplegia brace during the transfer motion to simulate motor impairment. The eight inexperienced participants had no caregiving experience and an average age of 22.4 ± 1.6 years, height of 171.1 ± 6.2 cm, and weight of 59.9 ± 4.5 kg. All participants were male, six were right-handed and two were left-handed. The skilled caregivers included six licensed physical or occupational therapists (four males and two females) with an average age of 31.7 ± 3.2 years, height of 171.3 ± 6.0 cm, and weight of 62.0 ± 6.0 kg. All the caregivers were right-handed and had an average of 8.3 ± 3.2 years of experience. The experiments were approved by the Maebashi Institute of Technology Ethics Review Committee for Human Function Experiments, and written consent was obtained from all subjects before the start of the experiment (No. 21-0004, No. 22-002).

**TABLE 1:** Characteristics of Participants

	Care recipient (n = 1)	Inexperienced participants (n = 8)	PT/OT (n = 6)
Age (y/o)	24	22.4 ± 1.6	31.7 ± 3.2
Height (cm)	163.0	171.1 ± 6.2	171.3 ± 6.0
Weight (kg)	50	59.9 ± 4.5	62.0 ± 6.0
Gender	Male	Male (n = 8)	Male (n = 4) Female (n = 2)
Dominant	Right	Right (n = 6) Left (n = 2)	Right (n = 6)
Experience (years)	N/A	None	8.3 ± 3.2

mean ± standard deviation.

The experimental procedure involved seating the care recipient on a nursing bed with their feet on the floor, waiting for the caregiver to transfer them to a wheelchair. The caregiver, either from the inexperienced or skilled group, was asked to transfer the care recipient using their preferred method. Data were collected continuously during the transfer using the three-dimensional motion-capture system, force plate, and the embroidered body pressure and proximity sensor until the transfer was completed. Each caregiver performed three trials of the transfer. The inexperienced group received feedback on the safety of the transfer motion from an occupational therapist (Dr. Ken Kondo, co-author of this paper). The following instructions were delivered verbatim during the transfer. Steps 4 and 5 were repeated three times.

1. Transfer the patient from the nursing bed to the wheelchair. Once the patient is lifted from the care bed, move the patient from the care bed to the wheelchair in a single movement without changing the location of the patient.
2. The patient has braces on the right arm and leg and cannot move them. The patient can move their left arm and leg.
3. Once a caregiver performs the transfer care operation, Dr. Ken Kondo of Gunma Paz University, an occupational therapist, checks the safety of the operation. If there is a significant problem in the transfer-of-care movement, he will provide verbal advice.
  • Upon confirming the movement
4) Now, we will start the measurement. Please start the nursing-care operation.
  • After the movement is completed
5) Thank you for your cooperation.

#### 3) Comparative Analysis of Motion Capture, Force Plate, and Embroidered Sensor Data

We verified the data obtained by our system by comparing with the data measured by conventional motion analysis devices, which included a force plate and a motion capture system. In our system, we measured the seat position and seating velocity in transfer motion.

**FIGURE 8.**
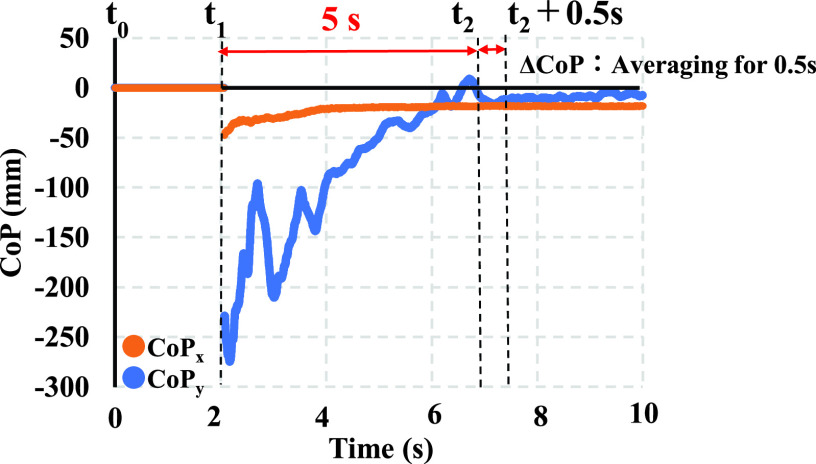
Example of change in CoP.

To verify the seating position, the center of pressure (CoP) from the force plate was compared with the sensor output. The data measured by the force plate were processed directly by its software and recorded into PC 1. Fig. 8 displays an example of the CoP waveforms measured using the force plate placed beneath the wheelchair. The horizontal axis represents the time (in seconds), and the vertical axis represents $\Delta $CoP, the coordinate change of the pressure center. At the onset of the measurement at t = t_0_, a caregiver started the transfer motion from bed to wheelchair. Once a care recipient was touched, his/her buttock was placed at the seat on the wheelchair at t = t_1_, and CoP measured by the force plate fluctuated over the seating phase. Finally, the care giver completed the transfer motion, and the CoP data became stable at t = t_2_. In this study, we defined time t_2_ after 5 (s) from the onset of being seated at t = t_1_. We determined the variability of CoP ( $\Delta $CoP) based on the data points between the initial and stable states. The average value of the data for t_2_ < t < t_2_ + 0.5 seconds was calculated as an index of the obtained seating position of the patient.

**FIGURE 9.**
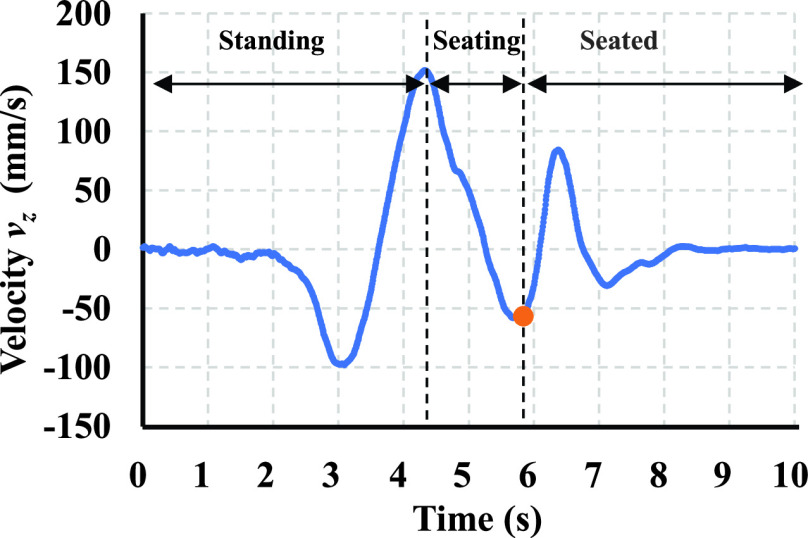
Seating velocity $v _{z}$ defined as the third peak during transfer from bed to wheelchair.

To verify the seating velocity, the motion velocity was obtained by the motion capture system and compared with the system output. Fig. 9 shows an example of the head motion velocity along the z-axis of a caregiver measured using the three-dimensional motion-capture system. Three distinct phases were observed: standing, sitting, and seated.

We sampled the negative peak velocity during the seating phase. In this study, the seating velocity $v_{z} $ (mm/s) is defined as the seating velocity measured by the marker attached on the parietal. We summarized all calculated parameters in Table 2. We attempted to find a relationship between CoG and $\Delta $CoP to verify the seating position, and a relationship between $\alpha $ and $v_{\mathrm {z}}$ to verify the seating velocity.

**TABLE 2:** Parameters Used in the Experiment

	Our system	Motion sensor device
Position	CoG	$\Delta $CoP from the force plate
Velocity	$\alpha $	$vz$ from motion capture

## III. Results

### A. Comparison of Seating Position Between Inexperienced and PT/OT Groups

**FIGURE 10.**
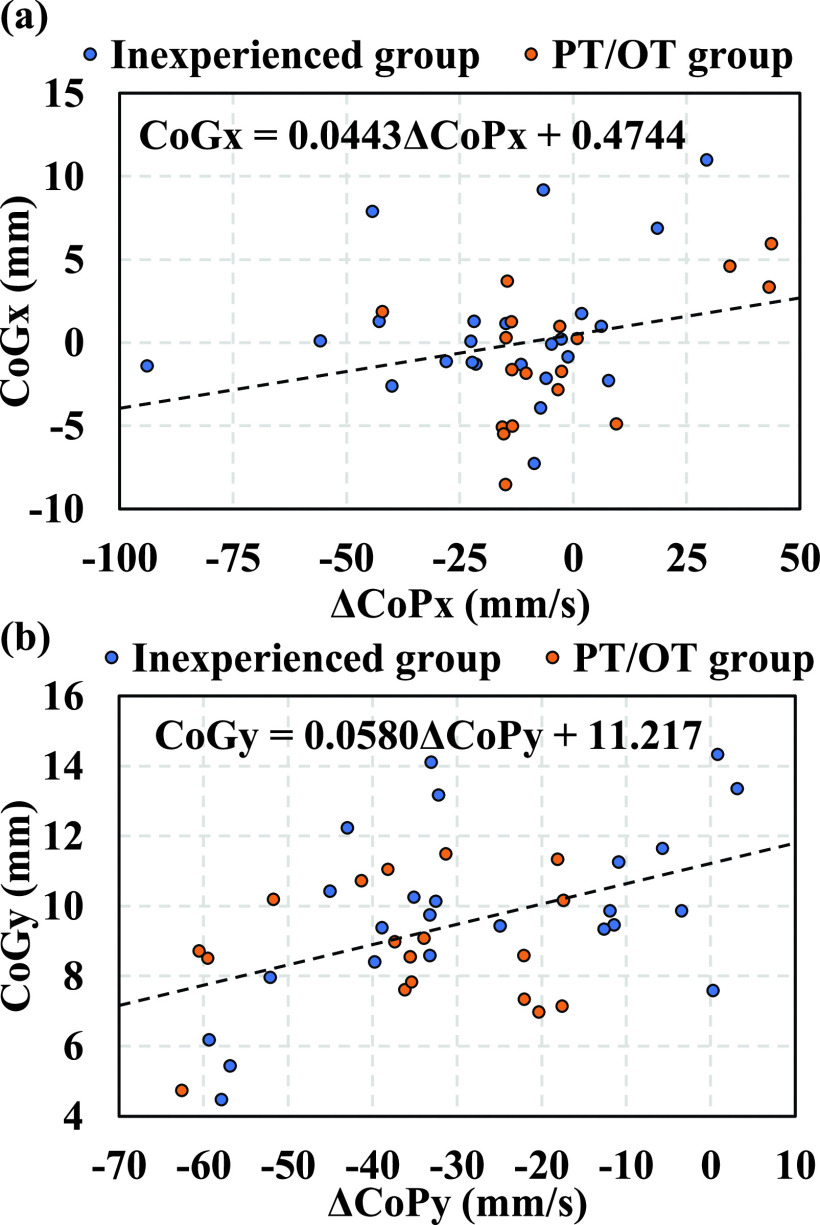
(a) Correlation between $\Delta $CoP and CoG (Total number of plots: N = 42. The inexperienced group: 24 points, the PT/OT group 18 points). (b) Result of simple regression analysis between $\Delta $CoP_y_ vs. CoG_y_ shows p-value < 0.01, t-value = 3.28. Correlation coefficient r = 0.46.

The relationships between the changes in the center of pressure ( $\Delta $CoP_x_, $\Delta $CoP_y_) recorded by the force plate and the centers of gravity (CoG_x_, CoG_y_) recorded by our sensor in the inexperienced and PT/OT groups were analyzed. Fig. 10 (a) shows the correlation between $\Delta $CoP_x_ and CoG_x_ and Fig. 10 (b) depicts the correlation between $\Delta $CoP_y_ and CoG_y_. The horizontal axis represents $\Delta $CoP (mm/s), and the vertical axis represents CoG. In these figures, the total number of points is 42, indicating three trials with eight and six participants in the inexperienced and PT/OT groups, respectively. Regression analysis was performed with $\Delta $CoP as the explanatory variable and CoG as the response variable. The results reveal that the explanatory variable $\Delta $CoP_y_ significantly affected the response variable CoG_y_(p-value < 0.01 and, t-value = 3.28), and the correlation coefficient r was found to be 0.46, indicating that CoG_y_ can reflect the seating position in the anterior-posterior direction. However, the correlation between CoG_x_ and $\Delta $CoP_x_ is not as strong, with a p-value and t-value of 0.083 and 1.78, respectively. The $\Delta $CoP_x_ values of the inexperienced group range from −93 to 29 mm, while that of the PT/OT group range from −42 to 43 mm. The $\Delta $CoP_y_ values range from −59 to 3.2 mm for the inexperienced group, and from −63 to −17 mm for the PT/OT group.

**FIGURE 11.**
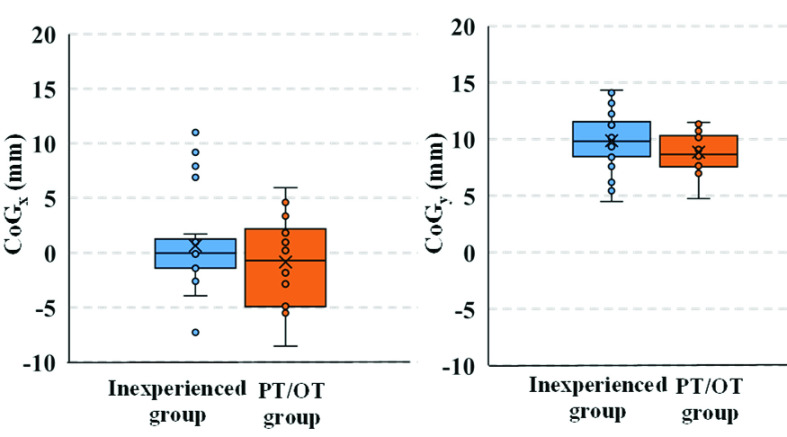
CoG of the inexperienced and PT/OT groups. Numbers of participants in the inexperienced and the PT/OT groups were 8 and 6, respectively.

In Fig. 11, a box-and-whisker plot demonstrates the variation in the CoG between the inexperienced and PT/OT groups. As depicted in the figure, the inexperienced group has several extreme CoG_x_ values, while the PT/OT group do not have any outliers in the CoG_x_ value. Moreover, the spread of the data in the CoG box-and-whisker plot for the inexperienced group is larger than that for the PT/OT group.

### B. Comparison of Seating Velocity Between Inexperienced and PT/OT Groups

**FIGURE 12.**
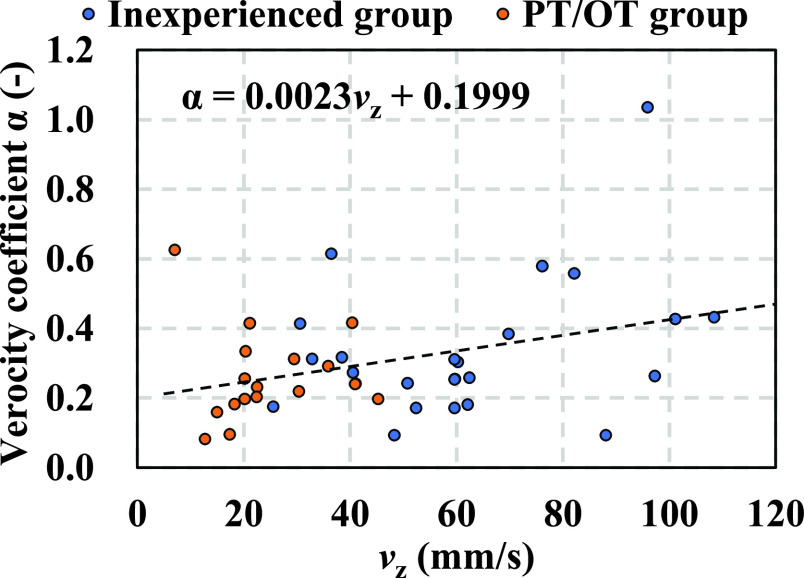
Correlation between $v _{z}$ and $\alpha $ (Total number of plots: N = 42. The inexperienced group: 24 points, the PT/OT group: 18 points). The result of simple regression analysis between $v _{z}$ and $\alpha $ shows p-value < 0.05, t-value = 2.24. Correlation coefficient r = 0.33.

**FIGURE 13.**
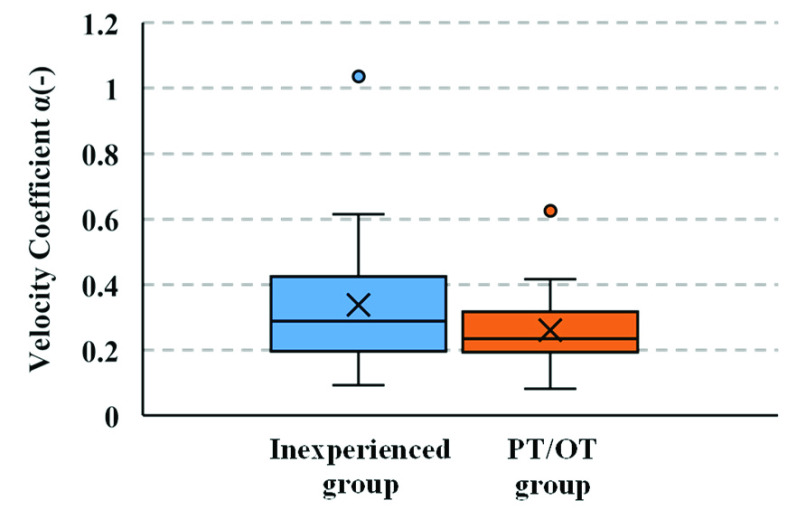
Velocity coefficient $\alpha $ of the inexperienced and PT/OT groups (based on the number of participants in the inexperienced and the PT/OT).

Fig. 12 illustrates the relationship between the velocity of seating ( $v_{z}$) measured using the three-dimensional motion-capture system and the velocity coefficient ( $\alpha$) calculated from the body pressure and proximity sensors. The results reveal that the explanatory variable, the actual seating velocity ${v} _{z}$ significantly affected the response variable, the velocity coefficient $\alpha $ (p-value < 0.05, t-value = 2.24). The correlation coefficient ${r}$ was found to be 0.33. The seating velocities of the inexperienced group ranged from 25 to 110 mm/s) while those of the PT/OT group ranged from 8 to 45 mm/s. Fig. 13 shows a box-and-whisker plot of the distribution of $\alpha $ for the inexperienced and PT/OT groups. The vertical axis represents the value of $\alpha $. As shown in Fig. 13, the value of $\alpha $ is lower in the PT/OT group than that in the inexperienced group. The results of the Mann-Whitney test exhibit a significant difference in the seating velocity $v_{z}$ of the inexperienced and PT/OT groups (p-value < 0.01).

## IV. Discussions

The box-and-whisker plot of the center of gravity (CoG_x_) in Fig. 11 suggests that the participants in the PT/OT group maintained a more balanced posture, primarily because there are no data points outside the top of the whisker. Conversely, the inexperienced participant group have data points beyond the top of the whisker, which can be attributed to the right tilt caused by the right paraplegia brace worn by the patient. Furthermore, the median value of CoG_y_ is higher in the inexperienced group (9.84 mm) than that in the PT/OT group (8.82 mm), and the third quartile is also higher in the inexperienced group (11.54 mm) than that in the PT/OT group (10.31 mm), indicating that the PT/OT group could seat the care recipient deeper in the chair than the inexperienced group.

The PT/OT group could seat the care recipient closer to the center of the seat than the inexperienced caregivers, and the differences in transfer care skills are indicated by the seating position of the body on the seat. The average value of CoG_y_ in the PT/OT group is slightly smaller than that of the inexperienced group. This indicates that the PT/OT intended to seat the care recipient’s buttocks inwardly and deeply to avoid a slouched posture which can cause shearing type pressure ulcers [11]. Therefore, the results suggest that the sensor response evaluation values of CoG_x_ and CoG_y_ can be utilized to distinguish between caregiving skills associated with different seating positions.

In the box-and-whisker plot of the velocity coefficient $\alpha $ in Fig. 13, one data point deviates beyond the top of the whisker for both datasets from the inexperienced and PT/OT groups. However, the overall velocity coefficient $\alpha $ in the PT/OT group tends to be smaller than that in the inexperienced group, and the median value of $\alpha $ in the inexperienced group (0.34) is larger than that in the PT/OT group (0.26). The third quartile is also higher in the inexperienced group (0.42) than in the PT/OT group (0.30), suggesting that the PT/OT group supported and seated the patient adequately. The velocity coefficient ( $\alpha$) was found to be correlated with the seating velocity and higher in inexperienced caregivers than in the PT/OT group, indicating that the inexperienced caregivers could not seat the care recipient slowly.

Iwakiri et al. [15] evaluated the lifting and lowering velocities while using equipment to lift a patient for transfer during nursing care. They conducted an experiment to evaluate the appropriate transfer velocity using a lifting device and measuring the HR and EMG. The recommended velocity for lowering the resident is 50 mm/s in the range of 30 to 90 mm/s.

In Fig. 12, the peak velocity ( $v_{\mathrm {z}}$) in the PT/OT group tends to be smaller than 50 mm/s. The velocity in the PT/OT group is appropriate, which aligns with the results of the aforementioned studies. Therefore, the velocity coefficient $\alpha $ can be used as an indicator to evaluate the differences in caregiving skills based on the seating velocity information. Accordingly, the system can serve as an alternative to the three-dimensional motion-capture system, acceleration sensors, and force plate sensors for analyzing caregiving motions.

## V. Conclusion

Based on the results of this study, it can be concluded that the embroidered capacitive pressure and proximity sensors are useful tools for evaluating transfer caregiving skills. The sensor response evaluation values and velocity coefficients could accurately distinguish between novice and experienced caregivers, suggesting that the proposed system can be employed to enhance the transfer care skills and provide a comprehensive evaluation of transfer caregiving.

Currently, the system is only applied to the seating phase of the transfer motion from a bed to a wheelchair, but it can also be used for the standing phase. Furthermore, the indicators CoG and $\alpha $ proposed in this study are applicable to other transfer training systems to provide feedback to the caregiver in the transfer of standing and seating phases in terms of the seating position and velocity.

In future work, we need to observe the difference in how an experienced caregiver performs transfers with/without the system. However, the current system does not have a real-time feedback function to provide sensor data to a caregiver. We will develop the next version with an online feedback function of transfer velocity and seating position. To determine the optimal and comfortable transfer velocity, it is necessary to conduct experiments that evaluate the physical burden during manual transfer by measuring the HR and EMG of the care recipient. Since the test subject in the study was a healthy male wearing a hemiplegia to simulate motor impairment, it is necessary to validate the effectiveness of the proposed system by involving actual patients with motor impairments. In other words, various types of disabilities in the evaluation of caregiver skills must be considered for better evaluation. Sensor output would differ depending on the body size of the patient and caregiver’s approach. Thus, various types of parameters must be considered to obtain more accurate skill evaluation results in transferring.
